# Identification of Two Commercial Pesticides by a Nanoparticle Gas-Sensing Array

**DOI:** 10.3390/s21175803

**Published:** 2021-08-28

**Authors:** Evangelos Skotadis, Aris Kanaris, Evangelos Aslanidis, Nikos Kalatzis, Fotis Chatzipapadopoulos, Nikolaos Marianos, Dimitris Tsoukalas

**Affiliations:** 1 Department of Applied Physics, National Technical University of Athens, 15780 Athens, Greece; ariskanaris@mail.ntua.gr (A.K.); evaaslani@central.ntua.gr (E.A.); dtsouk@central.ntua.gr (D.T.); 2 NEUROPUBLIC S.A., 18545 Piraeus, Greece; n_kalatzis@neuropublic.gr (N.K.); f_chatzipapadopoulos@neuropublic.gr (F.C.); n_marianos@neuropublic.gr (N.M.)

**Keywords:** gas sensors, chemiresistor, e-nose, smart farming, nanoparticles, chlorpyrifos, bupirimate, pesticide, vapour

## Abstract

This study presents the experimental testing of a gas-sensing array, for the detection of two commercially available pesticides (i.e., Chloract 48 EC and Nimrod), towards its eventual use along a commercial smart-farming system. The array is comprised of four distinctive sensing devices based on nanoparticles, each functionalized with a different gas-absorbing polymeric layer. As discussed herein, the sensing array is able to identify as well as quantify three gas-analytes, two pesticide solutions, and relative humidity, which acts as a reference analyte. All of the evaluation experiments were conducted in close to real-life conditions; specifically, the sensors response towards the three analytes was tested in three relative humidity backgrounds while the effect of temperature was also considered. The unique response patterns generated after the exposure of the sensing-array to the two gas-analytes were analyzed using the common statistical analysis tool Principal Component Analysis (PCA). The sensing array, being compact, low-cost, and highly sensitive, can be easily integrated with pre-existing crop-monitoring solutions. Given that there are limited reports for effective pesticide gas-sensing solutions, the proposed gas-sensing technology would significantly upgrade the added-value of the integrated system, providing it with unique advantages.

## 1. Introduction

In the field of pesticide detection, pollutants are usually identified and quantified via analytical methods and techniques such as chromatographic and coupled chromatographic-spectrometric procedures [1,2] or electroanalytical/voltammetric techniques [3]; bupirimate as well as chlorpyrifos are usually quantified using all aforementioned methods. The need, however, for fast, remote, and automated in-the-field determination of pesticides is increasing. Miniaturized, cost-effective, low-power, and sensitive sensors are in high demand, with the main technologies in the field of pesticide detection being Electrochemical, optical, and piezoelectric biosensors [4,5,6] as well as molecularly imprinted polymer sensors [7]. Gas-sensing of environmental pollutants is also on the rise since gas-sensors in general fulfil all of the aforementioned criteria by usually being low cost, compact, accurate, fast, and able to simultaneously determine multiple gas-analytes, and, contrary to most biosensors, they show reversible responses to their gas-target, thus making them reusable. The majority of the available solutions for gas-sensing of environmental pollutants deal with metal oxide sensors, which typically operate in elevated temperatures. Many solutions have been proposed if the field of micro and Nano gas-sensors in particular, where different nanomaterials have been discussed as potential active materials for gas-sensing. Sensors employing metal oxide semiconductors (MOS) usually rely on p-type or n-type MOS materials fabricated in the nanoscale [8]. Sensors based on Carbon Nanotubes (CNT) are a common alternative for the gas-sensing of NH_3_, NO_2_, CO, CO_2_ etc. [9]. Semiconducting materials in the nanoscale have been also used extensively for chemical sensing [10]; zinc oxide nanowires have been used for hydrogen sulphide detection (H_2_S), while the addition of catalytic Pt NPs further improved sensitivity and reduced the operating temperature of the device [11]. The addition of noble metal NPs is a common strategy that is employed to improve sensitivity and produce tailor-made chemical sensors. More recently, the integration of two different catalytic materials in the same device has been proposed to take advantage of the properties of both materials [12,13]. However, developing appropriate strategies for improving the selectivity of metal oxide (MOX)-based sensors remains a challenge; towards this direction, the incorporation of metal organic frameworks can improve the selectivity of MOXs sensors [14]. It is also worth noting that nanomaterials such as CNTs and MOXs, and organic materials such as conducting polymers, have been also used in the development of e-nose systems for demanding applications such as gas-sensing trace amounts of explosives [15].

Organic-based chemiresistive gas sensors are also of great importance in the field of electrochemical sensors. Such sensors usually rely on organic materials such as polymers [16], and their operation requires no additional heating or any elaborate/costly fabrication techniques. Apart from being cost-effective, their facile fabrication means that they can be easily combined with inorganic nanomaterials like metallic Nanoparticles (NPs). Their combination produces advanced, hybrid devices that can combine the organic layer’s high affinity and susceptibility towards volatile organic compounds (VOCs) and the high sensitivity of the inorganic layer towards small changes in its environment. This results in hybrid devices with improved properties such as higher sensitivity, lower limits of detection, and low-power requirements (NP based devices can be operated in voltages equal to or lower than 1 V, corresponding to a device current in the order of nA or μA), resulting in cost-effective and environmentally efficient devices (using extremely small amounts of materials) that are compatible with standard microelectronic processes and batch fabrication. Selectivity remains a challenging issue for polymeric materials; a common solution to surpass material related limitations is the post-processing of sensory data with statistical tools such as principal component analysis (PCA). In addition, device parameters such as accuracy, precision, reversibility, resolution, and response/relaxation time are also positively influenced by the hybrid nature of the devices with improved properties. Cross-linked Au NPs in particular have been widely used in order to produce chemiresistors capable of detecting various environmental pollutants [17] as well as VOCs for diagnosing diseases, such as the recent COVID-19 virus [18]. Up to now, there are scarce reports regarding pesticide gas-sensors, since most efforts usually focus on the electrochemical sensing of pesticides [19]. Dimethyl-methylphosphonate (DMMP, lab simulant of the toxic agent of sarin) has been detected with a limit of detection (LOD) in the ppm range, using complicated to produce single-use sensors [20]. Organophosphate vapour sensing via peptide nanotube biosensors with a short life span (45 days) and a LOD in the ppb regime has been also reported [21], and a SnO_2_ organophosphorus gas sensor operating at 250–300 °C with a LOD of 1.053 μg/L has been also discussed [22]. Finally a nanopore sensor (agarose gel-based chip) has been used for the detection of vaporized omethoate (LOD of 100 ppb) through the complex formation with a DNA aptamer, and its obstruction at the nanopore [23]. 

In the current paper, a gas-sensing array comprised of platinum (Pt) NP films covered by polymeric films is proposed as an “e-nose” system for the room-temperature detection of two commercially available pesticides, Nimrod and Chloract 48 EC. The sensors utilize previous experience of this group in the development and optimization of NP based gas-sensing devices for the detection of humidity, VOCs [24,25,26,27], and pesticides [28]. Up to now, the array-optimization process has mainly focused on enhancing device performance by improving its design as well as its fabrication process. The current study highlights the effect of environmental conditions such as background R.H. and temperature on sensor performance and ultimately proposes an appropriate scheme for the detection, identification, and quantification of two commercially available pesticides (Nimrod and Chloract 48 EC) over the ever-present in any farming application R.H. In addition, the current study highlights the challenges that the proposed system should overcome, towards its implementation in the field. Previous attempts in detecting pesticide vapours have resulted in successful active ingredient identification (chlorpyrifos) over its solvent for a laboratory prepared standard solution [28]. Results discussed herein showcase the capacity of the array in detecting and differentiating between all three gas-analytes (two of which are commercial pesticides), by optimizing environmental conditions such as R.H. and temperature and employing a common statistical analysis tool (i.e., principal component analysis (PCA)). In addition, instead of initializing and operating the sensors in a close to “zero-humidity” environment as in previously reported results [24,25,26,27,28], the sensors are now tested in elevated R.H., in an effort to facilitate their incorporation in commercial crop-monitoring systems for actual in-the-field applications. The hybrid polymer/nanomaterial-based device is ultimately optimized towards the detection of 2 commercially available pesticides at room temperature, proving that it can successfully identify and quantify between three gas targets; the proposed gas-sensing technology could therefore evolve to an e-nose system capable of separating between an expanded number of available gas targets and environmental pollutants. It’s also worth noting that, to the authors’ knowledge, there are no reports on the gas detection of any of the two commercially available pesticides, while, as far as Nimrod and bupirimate are concerned (Nimrod’s active ingredient), there are no reports on its detection, regardless of the choice in sensing technology (analytical methods aside). Given the overall scarcity of available publications in the field of pesticide gas sensing as well as the array’s important advantages related to competing gas-sensing technologies (reusable, fast, reversible, highly sensitive, cost-effective, low-power, and reliable), the proposed device is a unique solution that is also suitable for easy integration in commercial, advanced, and remote monitoring systems. 

## 2. Materials and Methods

The chemical sensing array combines two-dimensional (2D) conductive metallic nanoparticle (NP) films and gas-sensitive polymeric films. The NP layer of the device is responsible for its conductivity while the polymer films, being susceptible to volatile organic compounds (VOCs), act as the gas-sensitive layer. Device fabrication begins by patterning oxidized silicon substrates with gold interdigitated electrodes (IDEs). IDEs are patterned by employing photolithography and the e-gun deposition of 5 nm of Ti (acting as an adhesion layer) and 30 nm of gold; IDEs inter-finger spacing has been selected to be 10 μm. As a second step, Pt NPs are deposited and fabricated in a single step on top of the silicon substrates, utilizing the DC sputtering technique, as described in previous publications by this group [24,26]. The surface coverage of the devices reported in the current paper has been selected for optimized device-sensitivity (devices with a mean resistance in the range of 700 kΩ). It is important to note that all fabrication steps were performed at room temperature (25 °C). Device fabrication was finalized by spin-coating 4 distinctive polymers on top of the Pt functionalised substrates. Polymeric solutions were prepared using the following materials that were bought from Merck: poly (ethyl methacrylate) (PEMA), poly (2-hydroxyethyl methacrylate) (PHEMA), poly (isobutyl methacrylate) (PIBMA), and poly (butyl methacrylate) (PBMA). The selection of the polymeric materials was based on their performance regarding the detection of a laboratory synthesized Chlorpyrifos solution [28]. All polymers were bought as powders and have been fabricated using a high precision micro-balance; PGMEA and methanol have been used as solvents for PEMA, PIBMA, PBMA, and PHEMA, respectively. As a final step, the fabricated polymeric solutions were spin-coated on top of the device-substrates in order to obtain a polymeric film of 500 nm in thickness. Film thickness has been confirmed via profiling measurements.

Two commercially available pesticides were purchased from a local agricultural supply store and used as target gases in the current study: Chloract 48 EC and Nimrod. Chloract 48 EC solution, a chlorpyrifos (CPS) based insecticide, was used according to its respective guidelines; for general use in crops, it is recommended that 100–250 c.c. of Chloract should be used per acre. It is also recommended that 65 L of Chloract solution (with water as a solvent) must be used per acre. All experiments in the current paper used a Chloract test-solution that was fabricated as follows: 175 c.c. of Chloract in 65 L of water. Our finalized Chloract-test solutions were 50 mL in volume, containing 0.081 c.c. of Chloract; this results in a Chloract test-solution composition of 0.12% CPS, 0.15% auxiliary substances, and 99.73% water (according to the manufacturer’s documentation). Nimrod solutions (a bupirimate-based fungicide) were also prepared according to the manufacturer’s guidelines; specifically, 250 c.c. of Nimrod should be used for 100 L of Nimrod/water solution, while 75 L of the finalised Nimrod/water solution should be used per acre. A Nimrod solution of 50 mL was used throughout the following experiments, corresponding to 0.125 c.c. of Nimrod. According to Nimrod documentation, its actual synthesis corresponds to 27.5% bupirimate, while the rest corresponds to auxiliary substances. This translates to a solution that is 0.069% bupirimate, 0.18% auxiliary substances, and 99.75% H_2_O in concentration.

**Figure 1:**
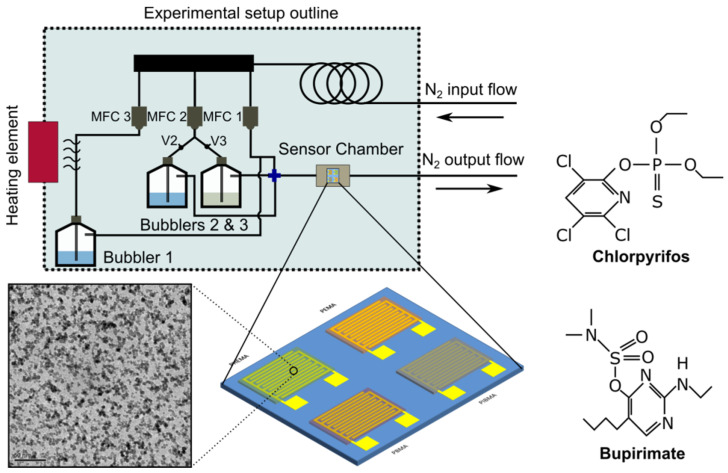
Schematic of the experimental gas-sensor characterization setup, for the detection of Chlorpyrifos and Bupirimate (chemical structure as shown herein). Three mass flow controllers (MFCs) are responsible for distributing the carrier-gas (N_2_) within the system. The MFCs are controlled via a PC and a Labview program; the same PC records the output (resistance) of the sensors via a home-made software. A schematic of the four interdigitated electrodes functionalised with four distinctive thin-polymer films (gas-sensing array), as well as a TEM image of the Pt NP film, are also included.

All gas-sensing experiments were conducted in the gas-sensing characterization setup that is available at the School of Applied Physics of the NTUA; a schematic of the specific setup used for the gas-sensing experiments is provided in Figure 1. Notably, the sensors are exposed to varying concentrations of one of the two pesticides/analytes vs. humidity. Specifically, 50 mL of either Chloract or Nimrod test-solutions were placed inside Bubbler 3, while 50 mL of DI water (reference analyte) were placed inside bubbler 2. Their respective vapours were collected by feeding the bubblers with a separate Nitrogen gas-line, externally controlled by two Nitrogen Mass Flow Controllers (MFCs). The Nitrogen gas-flow can be directed in either bubbler (2 or 3), by opening the respective valves (V2 and V3), as demonstrated in Figure 1. Chlorpyrifos or bupirimate solutions were prepared according to the manufacturer’s guidelines; the composite gas-mixture that is eventually introduced inside the sensing-array’s measuring chamber has a composition that depends on the partial vapour pressures of each solution-ingredient (water, auxiliary substances, and chlorpyrifos or bupirimate). In order to calculate the actual concentration of each active-ingredient (chlorpyrifos or bupirimate), Raoult’s law was employed (a good approximation for dilute solutions). In order to apply Raoult’s law effectively, the partial vapour pressure of each substance inside the water-mixtures must be known. However, in the case of proprietary commercial pesticides, there is a great number of unknown auxiliary substances whose vapour pressure needs to be approximated; herein, we have approximated the vapour pressure of the auxiliary substances with p-xylene, which is a common solute for pesticides [29]. Varying concentrations of Chloract, Nimrod, or humidity (corresponding to varying Nitrogen flow-rates) were superimposed over a continuous background-flow of nitrogen and humidity (originating from Bubbler 1), which eventually flowed inside the sensors’ measurement chamber. The purpose of the background-flow is to create a stable, artificial atmosphere of constant relative humidity (R.H.) so as to record the performance of the sensing-array in realistic conditions. R.H. is an ever-present “background” gas in any greenhouse and farming environment; any sensing-array that aspires to successfully detect pesticides and other environmental pollutants in the gaseous state should be able to distinguish such pollutants from any other gas over a constant background value of environmental R.H. 

For all experimental results reported in this paper, a comparison of the sensing-array’s response between Chloract, Nimrod, and humidity has been conducted. This comparison was for identical carrier-gas (Nitrogen) flows. For the sensors’ packaging, a small volume plastic chamber was 3D printed, ensuring efficient isolation between the sensing array and its environment; ultimately, R.H. was controlled within 0.1% and temperature within 0.1 °C. A commercial sensor was also used to track R.H. and temperature inside the array’s packaging, while the array’s resistance was monitored using a Keithley 2400 multimeter. Finally, that identical nitrogen flows of each pesticide and R.H. were introduced successively and in alternating order inside the sensing-array chamber (e.g., the introduction of 32 mL/min of a pesticide analyte would be followed by the introduction of 32 mL/min of R.H. or vice versa).

## 3. Results and Discussion

The idea behind hybrid NP/polymer-based sensors has been discussed extensively by this group in previous publications, where humidity and other VOCs (including CPS) have been successfully detected [24,25,26,27,28]. Furthermore, the sensors have been optimized in relation to all available fabrication parameters (i.e., IDEs geometry, NP surface coverage, device conductivity etc.); the devices discussed in the current paper were fabricated according to previous optimization studies [24,26]. Such sensors rely on the polymer layer for interacting with any VOC in the vicinity of the sensor; this interaction in turn modifies the conductivity (or resistance) of the NP layer. The four polymers selected in the current study are susceptible to most VOC and partially selective; by absorbing gaseous compounds, the polymer films begin to swell and therefore induce a strain in the underlying NP layer. This strain ultimately translates to an increase in the average inter-particle distance and, hence, an increase in the resistance of the device. One must bear in mind that the poor selectivity of all polymer coatings is an inherent disadvantage that is prominent when sensing composite gas mixtures. This partial selectivity leads to increased confusion over the identity of the measured gas target. However, such obstacles can usually be surpassed by employing statistical analysis techniques such as principal component analysis (PCA), which is commonly used for analysing gas-sensing responses for composite gas mixtures [30,31].

Previous publications regarding the detection of CPS vapours have focused on an analytical approach that investigated the sensors’ ability to detect the active ingredient (namely CPS) of a laboratory-synthesized, CPS based standard solution [28]. More recent results have reported on the detection of a commercially available CPS product [29], where the sensors have been always tested in a laboratory “zero-humidity” environment. In that case, a constant background Nitrogen flow of 1000 mL/min was applied inside the gas-measuring chamber, keeping R.H. close to 0% according to a commercial R.H. sensor; any other target-analyte had to be superimposed on-top of this constant Nitrogen flow. 

On the other hand, results discussed herein show the response of the sensing array in close to actual in-the-field conditions, where R.H. is not close to a “zero-humidity” environment. This especially holds true for greenhouse applications as well as in open crops. The sensors were initially tested in four different R.H. backgrounds (10%, 20%, 30%, and 40%). After calibrating the sensors towards background R.H., the sensing-array was submitted to a constant R.H. background and introduced to a range of varying concentrations of two commercially available pesticides (Chloract 48 EC and Nimrod) with different active ingredients (CPS and bupirimate, respectively). Each of the two pesticides has been also compared to varying concentration of R.H. (by successively feeding bubblers 2 and 3 with identical Nitrogen flows, as illustrated in Figure 1); specifically, the nitrogen flow-rate values that were employed to achieve R.H. concentrations in the range of 50–80% were also used in the case of Chloract and Nimrod solutions. In addition to the above, the sensors have also been tested in three different temperatures (20, 25, and 30 °C) so as to investigate the effect of temperature in sensor performance. For all experiments discussed below, twenty sensors have been employed in total (five for each distinctive polymeric coating).

### 3.1. Effect of Background Humidity on Sensor Response 

**Figure 2:**
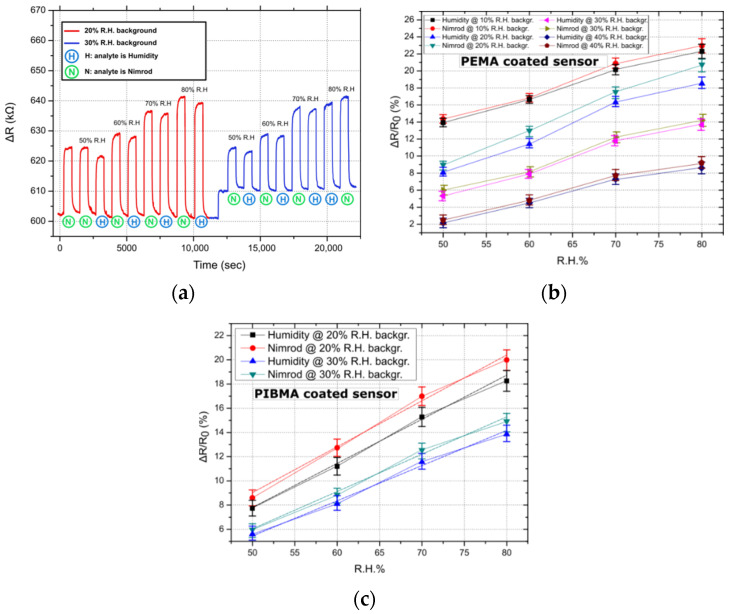
Typical sensor responses for PEMA and PIBMA coated sensors. Linear fitting was performed in the case of the best performing sensor (i.e., PIBMA sensors), to showcase the linearity of the sensors’ response. (**a**) Transient sensor response (ΔR) for the entire range of measured concentrations: 50–80% of R.H., for a single PEMA sensor. Nimrod measurements correspond to flow rates (measured in ml/min) identical to those used to achieve R.H. values from 50% to 80%. Results are typical of the response of all four polymer coatings, namely PEMA, PHEMA, PIBMA, and PBMA. (**b**) Relative resistance response (measured as ΔR/R0) for PEMA coated sensors and in four different R.H. backgrounds (backgr.). Data points correspond to the mean response of five sensors, while standard deviations are presented as error bars (from 0.56% to 0.87%). (**c**) Typical relative resistance response for PIBMA sensors, focusing on 2 R.H. backgrounds (20% and 30%). Data points correspond to the mean response of five sensors, while standard deviations are presented as error bars (0.61% to 0.94%). Linear fitting was performed and is presented as dashed lines. The adjusted R-square value for the linear fit regression was found to be between 0.98926 and 0.99559.

In order to study the effect of background humidity on sensor response, the sensing-array was tested in four different humidity backgrounds: 10%, 20%, 30%, and 40% of R.H; the temperature was kept constant at 25 °C. It has been suggested in previous publications that polymer-based R.H. chemo-resistors should incorporate polymers that are insoluble in water but sensitive to humidity [32]; this can be achieved through graft-polymerization of porous polymer films or through the formation of an interpenetrating polymer network of cross-linked hydrophilic and hydrophobic polymers. The degree of cross-linking can also play an integral role in sensor resistance towards water and its overall performance [33]; by using appropriate solutes and cross-linking polymer films in elevated temperatures, durable and robust humidity sensors can be produced. For each of the four R.H. backgrounds, the nitrogen gas flows feeding the pesticide or the DI water bubbler were selected so that, when combined with the background R.H., a total R.H. of 50%, 60%, 70%, and 80% would be achieved. For this series of experiments, only one of the two gas target-analytes, namely Nimrod, was used. In Figure 2a, the transient sensing response of a single PEMA coated sensor in Nimrod and R.H, for 20% and 30% R.H. backgrounds, is presented; the sensor’s response is typical, resembling the response of PHEMA, PBMA, and PIBMA coated sensors. For all polymeric coatings, the sensors systematically demonstrate an increased response towards the Nimrod solution. In Figure 2b,c, the ΔR/R_0_ (%) response of all 5 PEMA sensors in Nimrod and R.H., for 4 different R.H. backgrounds, is presented. By increasing the R.H. background, the response of the gas-sensing array for both pesticide analytes (measured as ΔR/R_0_%, where R_0_ is the resistance of the sensor prior to its exposure to any gas target concentration) is reduced. Figure 2a clearly demonstrates that, by increasing the background R.H. from 20% to 30%, the baseline resistance of the sensors is increased; this in turn translates to a reduced response to any of the gas analytes. The change in R.H. background also affects the sensors’ differentiation between Nimrod and R.H., making the R.H. background of 20% the one for maximum separation (Figure 2b,c). It is envisioned that the eventual integration of the sensing-array in a smart-farming tool will be accompanied by commercial humidity sensors as well as air-drying solutions (e.g., membrane air drying, vacuum drying etc.) in order to control ambient conditions inside the array’s packaging. Air drying solutions should be examined in parallel with the packaging development of the array. A feedback loop that will monitor the R.H. inside the packaging via commercial R.H. sensors will be able to provide an environment of 20% R.H. by mixing ambient air with partially dried air. 

### 3.2. Effect of Temperature in Sensor Response

**Figure 3:**
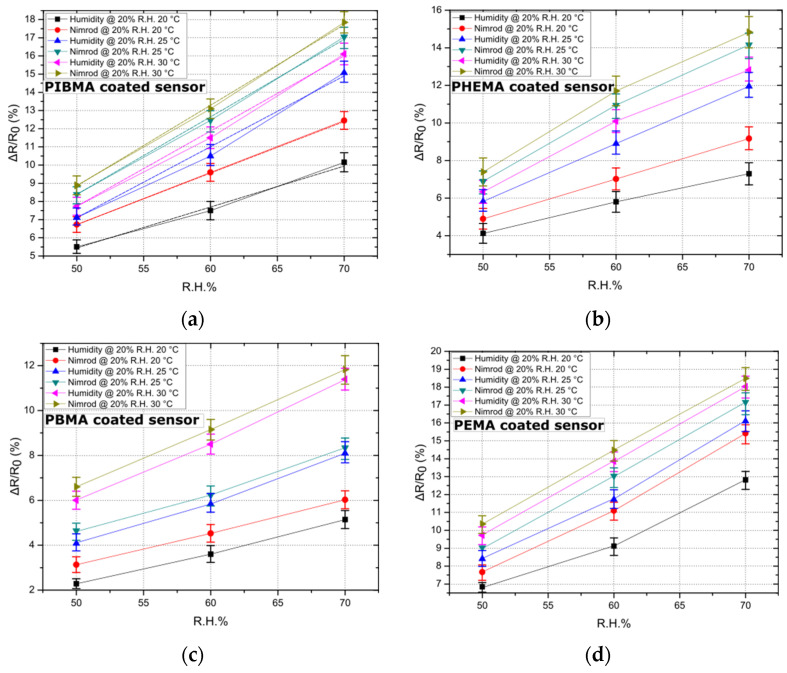
The effect of temperature on sensor response. Data points correspond to the mean response of five sensors (coated with each of the four polymers), while the standard deviation is illustrated by error bars. Sensors have been exposed to three temperatures: 20, 25, and 30 °C, while linear fitting has been performed in the case of the best performing sensor (i.e., sensors functionalized with PIBMA polymer) to showcase the linearity of the sensors’ response. (**a**) Response of 5 PIBMA coated sensors in Nimrod and R.H.; standard deviation from 0.42% to 0.66%. Linear fitting has been performed and is illustrated by dashed lines. The adjusted R-square value for the linear fit regression was found to be between 0.98567 and 0.99999. (**b**) Response of 5 PHEMA coated sensors in Nimrod and R.H.; standard deviation from 0.58% to 0.86%. (**c**) Response of 5 PBMA coated sensors in Nimrod and R.H.; standard deviation from 0.25% to 0.71%. (**d**) Response of 5 PEMA coated sensors in Nimrod and R.H.; standard deviation from 0.26% to 0.67%.

As a second step, the effect of temperature on sensing performance has been studied. The sensing array has been exposed to humidity and one of the target analytes of the study (i.e., Nimrod) in three different temperatures: 20, 25, and 30 °C. It is worth noting that our experimental setup can provide temperatures between 20 and 30 °C. Results provided in paragraph 3.1 regarding the effect of ambient R.H. in sensing performance were also taken into account: background R.H. was adjusted to 20% throughout this study (maximum Nimrod to R.H. differentiation). Additionally, the DI water and Nimrod bubblers were fed with Nitrogen flows that are equivalent to R.H. values between 50% and 70%; the acquired data set was adequate so as to establish the effect of temperature on sensor response. As demonstrated in Figure 3, and for all polymeric coatings, a rise in temperature also signals a rise in sensitivity of the sensors. A change in the response of the sensors is to be expected since the nanoparticle-based sensors discussed in this study were designed for optimum sensitivity, which corresponds to specific nanoparticle surface coverage and therefore device resistance. Optimized devices feature nanoparticle surface coverage that lies just below the percolation threshold, while their conductivity is governed by a thermally activated tunneling mechanism [29]. A rise in temperature is expected to contribute to a drop in resistance, which was validated in the results discussed herein. However, the increase in sensitivity observed in Figure 3 should be mainly attributed to the polymeric coatings. Various reports indicate that polymers increase in terms of hole-free volume (volume stemming from volume relaxation and plasticization upon heating and cooling of the polymer and accessible for penetrant transport) for increasing temperature [34]; in addition, the diffusion coefficient for polymers increases for increasing temperature [35]. The overall increase in the swelling of the polymer coatings (temperature as well as vapour induced), as well as enhanced diffusion, translates to increased sensor response, in accordance with our already reported gas-sensing mechanism. The time needed for reaching 70% of equilibrium state (mean response time) for all 16 sensors, was 17 s. A quasi-linear response towards Nimrod was recorded for all sensors, while the minimum detectable change in R.H. gas analytes (sensors’ resolution) was 0.48% (or 150 ppm). Finally, experimental results suggest that for 20 °C the sensors exhibit maximum differentiation in terms of sensing response for the two analytes (Nimrod and humidity). Controlling the temperature of a small footprint sensing array (single sensor dimensions are 1 mm by 0.8 mm) can be achieved using thermoelectric modules [36]; such modules can operate as heat pumps, providing heating or cooling of an object connected to one side of a thermoelectric module if a DC current is applied to the module’s input terminals, and are widely used in MEMS technology.

### 3.3. NP-Based Sensing Array for Discerning between Chloract 48 EC, Nimrod, and R.H.

**Figure 4:**
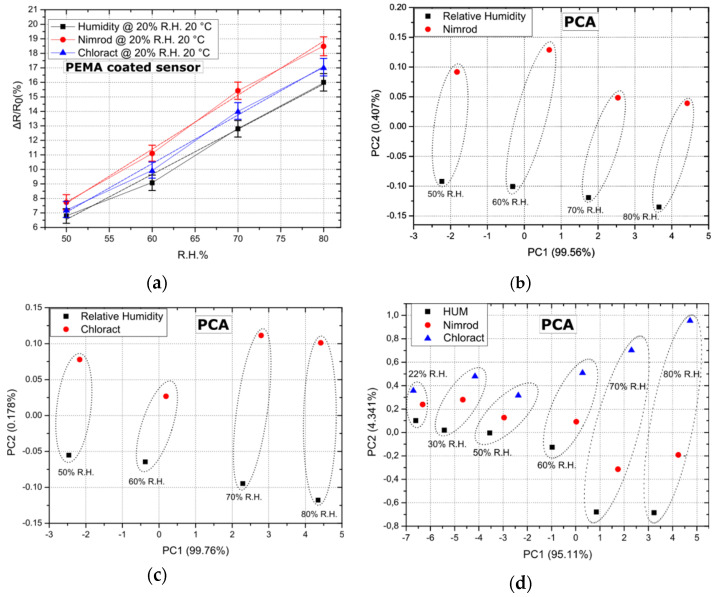
Sensor response and PCA for a fixed R.H. background of 20% and a temperature of 20 °C. Dotted lines (ellipses) have been used to group data points (**b**–**d**) that correspond to the array’s response for identical gas-flow rates for all three target-analytes. (**a**) Response of 5 PEMA coated sensors to R.H. (humidity), Nimrod and Chloract 48 EC. Error bars represent the standard deviation of the measurements between 0.495% and 0.71%. Response is typical of sensors coated with all other polymeric films, while linear fitting was performed and is represented as dashed lines. The adjusted R-square value for the linear fit regression was found to be between 0.98836 and 0.99206. (**b**) PCA for experimental data sets regarding Nimrod vs. relative humidity, for varying flow rates/concentrations. (**c**) PCA for experimental data sets regarding Chloract vs. relative humidity, for varying flow rates/concentrations. (**d**) PCA for experimental data sets regarding Nimrod vs. Chloract vs. relative humidity, for varying flow rates/concentrations. PC1 and PC2 percentages represent the amount of total information that is found in each of the two PCA components, as provided by the PCA.

As a final step, the sensing array has been examined as a potential “e-nose” for discerning between Nimrod, Chloract 48 EC, and R.H. The ability of the array to discern between CPS (Chloract’s active ingredient) and its solvent [21], as well as between a commercial CPS product and R.H. in a close to “zero R.H. environment” was demonstrated in the past by this group [29]. However, the ability of the array to discern between two commercially available pesticides and R.H. in elevated levels of background R.H. is an even more challenging task. In Figure 4a, the response of five PEMA coated sensors to Nimrod, Chloract 48 EC, and R.H. is presented; results correspond to a R.H. background of 20% and a temperature of 20 °C. Results for PEMA are typical for all other polymer coatings, exhibiting the highest sensitivity to Nimrod vapours, followed by Chloract 48 EC and R.H. Notably, the maximum separation between Chloract and Nimrod was achieved for the same conditions of 20% R.H. and 20 °C, while successful separation was also achieved between the three gas target analytes for a R.H. of 20% and temperatures of 25 and 30 °C.

The successful operation of a gas-sensing array as a potential e-nose system lies in the ability of the distinctive sensing devices to produce unique response patterns. The gas-sensitive elements of this device are the four polymeric coatings; each coating is characterized by its own unique diffusion rate, hence the swelling towards separate analytes [37]. In this way, unique gas sensing patterns are generated. However, the capability of the array to differentiate between various gas targets, as well as to quantify them, can only be validated by implementing statistical analysis tools or pattern recognition algorithms in the post-processing of the array’s read-out. Principal component analysis (PCA) is a statistical tool commonly used to analyze the output of gas-sensing arrays [30,31] to discriminate between target analytes in a composite gas mixture. To that end, PCA has been applied over our existing data-set (array’s response to Nimrod, Chloract 48 EC, and R.H.), in an effort to validate its use as a potential “e-nose” system. In Figure 4, PCA results are provided after the array’s response to Chloract vs. R.H., Nimrod vs. R.H., as well as Chloract vs. Nimrod vs. R.H., respectively, always in the presence of a fixed R.H. background of 20% and for a temperature of 20 °C. PCA of the array’s response results, using only 2 or 3 distinctive polymeric coatings, was also executed in all possible combinations; this revealed that a necessary condition for successful PCA separation between R.H. and Chloract is to include in the PCA the response of PHEMA coated sensors, while in the case of Nimrod: PEMA coated sensors. PHEMA and PEMA must be included in the PCA for successful separation between the analytes when applying the method in data-sets originating from the response of two or three polymeric films. By including the response of all four polymeric coatings, PCA results in even better separation between the analytes; Figure 4 illustrates this by introducing three target analytes on top of the fixed R.H. background. The array can differentiate not only between gas targets of identical flow-rate (data sets within the same dotted ellipses) but also between varying flow rates (distinctive dotted ellipses); this is due to the fact that PCA data points do not overlap for identical or varying flow rates of the three target analytes. The need for including R.H. as an additional gas target in Figure 4 arises from the fact that, in a real-case scenario, the array must be able to distinguish between short-term R.H. increases (caused e.g., after plant watering) and the event of applying a water-based solution that contains pesticides. Finally, the minimum concentration for which the array can separate between the three analytes was also investigated. As illustrated in Figure 4d, the sensing array can successfully separate between Chloract, Nimrod, and R.H. for a flow rate that is no less than 3.4 mL/min, and is superimposed over a background R.H. of 20% (flow rate of 24.2 mL/min), corresponding to a total R.H of 22%, as illustrated in Figure 4d. This flow rate corresponds to a humidity concentration of 500 ppm (and a R.H. of 2%), a chlorpyrifos concentration of 10.9 ppb, and a bupirimate concentration of 9 ppb (approximated using Raoult’s law, as described in Section 2).

## 4. Conclusions

A hybrid gas-sensing array based on platinum nanoparticles and functionalized with four distinctive polymeric films, was tested as a potential “e-nose” for detecting, quantifying, and separating between two commercially available pesticides (Nimrod and Chloract 48 EC) and relative humidity (R.H.). The effect of environmental parameters such as background R.H. and temperature on the performance of the sensors was evaluated, identifying a background R.H. of 20% and a temperature of 20 °C as optimal conditions for maximum separation between R.H. and pesticides. As a final step, the response data obtained for exposing the array to all three analytes were post-processed using principal component analysis (PCA). PCA validated the array’s capacity for discriminating and identifying between the three test gases, as well as for successfully operating for the first time in an environment of elevated background R.H.

The importance of the results reported in the current study is further highlighted if the number of existing pesticide gas-sensing reports, as well as the detection of commercially available pesticides and bupirimate, are also taken into account. The proposed sensing array emerges as an appealing pesticide-sensing solution for seamless integration in portable and remote smart-farming monitoring tools [38], being low-cost, compact, low-power, highly sensitive, fast reversible/reusable, and reliable. Future plans include further investigation of the effect of environmental conditions on sensor response, investigation of possible solutions in order to control temperature and R.H. inside the gas-sensing chamber during measurements, the eventual integration of the proposed sensing technology with the “gaiasense” smart-farming tool [39], and its performance evaluation in an operational environment. Finally, the expansion of the number of potential environmental pollutants that can be identified by the array, as well as expanding the degrees of freedom of the system by adding more polymeric films, are amongst our future goals.

## Data Availability

The data presented in this study are available on request from the corresponding author. The data are not publicly available due to privacy restrictions.
